# Visual exploration in adults: Habituation, mere exposure, or optimal level of arousal?

**DOI:** 10.3758/s13420-021-00484-3

**Published:** 2021-09-20

**Authors:** Erik Gustafsson, Coralie Francoeur, Isabelle Blanchette, Sylvain Sirois

**Affiliations:** 1 Department of Psychology, University of Portsmouth, King Henry I Street, Portsmouth, PO1 2DY UK; 2 Département de Psychologie, Université du Québec à Trois-Rivières, Trois Rivières, QC Canada; 3 École de Psychologie, Université Laval, Québec, QC Canada

**Keywords:** Habituation, Mere exposure effect, Optimal-level of arousal, Engagement, Exploratory behaviours

## Abstract

Exploration is one of the most powerful behaviours that drive learning from infancy to adulthood. The aim of the current study was to examine the role of novelty and subjective preference in visual exploration. To do this, we combined a visual exploration task with a subjective evaluation task, presenting novel and familiar pictures. The first goal was to ascertain whether, as demonstrated in babies, short habituation favors visual exploration of familiarity, whereas longer habituation leads to an exploration of novelty. The second goal was to evaluate the influence of familiarization on participants’ subjective evaluation of the stimuli. When presented with novel and very familiar stimuli, participants explored the novel stimuli more. In line with the optimal-level of arousal model, participants showed more positive evaluations of the semi-familiar stimuli compared with very familiar or very novel ones.

Exploration is one of the most powerful behaviours that drive learning from infancy to adulthood (Oudeyer et al., 2016). Much current cognitive research explores the mechanisms of learning without considering how and why certain stimuli are prioritized for exploration (Mather, 2013). Yet the factors driving exploratory behaviours are strong determinants of what skills will be learned and subsequent developmental trajectories.

Habituation and mere exposure effects have been the main models used to explain the human response to novelty and familiarity. According to habituation theory, repeated exposure to a stimulus typically leads to a drop in interest towards that stimulus, allowing for novelty preference. Habituation is a simple form of nonassociative learning, in which a behavioral response decreases as a result of repeated stimulation, without involving sensory adaptation, sensory fatigue, or motor fatigue (Rankin et al., 2009). This form of learning has an adaptive value by allowing human and nonhuman animals to filter out irrelevant iterative elements to direct their attention to new stimuli (Eisenstein et al., 1995). Habituation has been shown in several studies with human adults, mostly for visual (Bernstein, 1969; Bradley et al., 1993; Hare et al., 1970; Mangelsdorff & Zuckerman, 1975) and auditory stimuli (Eisenstein et al., 1995; Potter et al., 2015).

The orientation response following habituation has mainly been studied in terms of physiological response to stimulus repetition, such as changes in electrodermal activity, heart rate, or event-related potentials (Bradley, 2009; Graham & Clifton, 1966; Sokolov, 1963). Several studies on covert orienting of attention have also used response times from the appearance of the target, to investigate habituation to distracting stimuli (Turatto et al., 2018; Turatto & Pascucci, 2016). However, behavioural responses such as looking time have received little attention in human adults. In contrast, looking time has been used extensively in infant research on habituation (Fantz, 1958; Oakes, 2010). For instance, once the infant is habituated to a stimulus or a class of stimuli (typically measured by a drop in looking time), researchers can examine which novel stimuli or stimulus features may produce renewed interest. This can allow testing discrimination abilities, and identify what infants perceive as novel relative to familiar (Sirois & Mareschal, 2004). In such habituation research with babies, the term “novelty preference” is often used when participants are looking relatively more (frequency and/or duration) at new stimuli (Fantz, 1958). This should not be construed as a positively valenced behavior, as preference in this case is related to preferential processing, and it distinguishes alternative behaviors purely quantitatively (relative looking times, relative changes in heart rate, etc.).

In contrast, the mere exposure effect is another psychological phenomenon that describes systematic valenced preference (a positive attitude) towards familiarity, not novelty. Zajonc (1968) proposed that the mere repeated exposure to a stimulus enhances a person’s attitude toward it. In one of his experiments, participants were exposed to nonsense words. Each word was presented 0, 1, 2, 5, 10, or 25 times. Results revealed that words with 5, 10, and 25 exposures were rated as more positive than the ones with 0, 1, or 2 exposures. The mere exposure effect has since been shown in several modalities: visual (Bornstein, 1989; Zajonc, 1968, 2001), auditory (Heingartner & Hall, 1974; Wilson, 1979), olfactory (Balogh & Porter, 1986; Cain & Johnson, 1978), and gustatory (Crandall, 1985; Pliner, 1982). According to the hedonic fluency model (Bornstein & D’Agostino, 1994), this preference for familiarity results from a more fluent processing of familiar stimuli (Clore & Huntsinger, 2007; Clore & Palmer, 2009; Reber et al., 2004; Rotteveel & Phaf, 2007; Schwarz et al., 1991; Whittlesea, 1993; Whittlesea & Williams, 2001; Winkielman et al., 2003).

Thus, there are seemingly contradictory results stemming from the habituation and the mere exposure literatures; the first facilitating orientation towards novel information in preference tests, while the latter results in orientation towards familiar information. Why would we explore stimuli we like less to a greater extent? Three theoretical models, the optimal-level of arousal (or stimulation) model, the two-factor model of mere exposure, and the dual-process theory provide explanations for this apparent contradiction. They all predict a preference for semi-familiarity, or semi-novelty (Berlyne, 1960, 1970; Bornstein, 1989; Colombo & Mitchell, 2009; Kaplan et al., 1990; Mather, 2013; Montoya et al., 2017). The first model hypothesizes that individuals seek and prefer an optimal (usually moderate) level of arousal. The second model conceptualizes the mere exposure effect in terms of the combined effects of habituation, which makes new stimuli easier to process and less threatening, and boredom, which results in the decline of positive affect. In the same vein, the dual-process theory predicts an *increase* in the strength of response towards familiar stimuli due to the sensitization attributable to a transient “spike” in arousal (which translates into an increase in exploration), followed by the decrease in response to the stimulus that characterized habituation and boredom.

Several studies in infants examined the effect of two types of habituation: short, incomplete habituation (habituation is partially induced), and longer, more complete habituation (i.e., when resulting in a substantial drop in behaviour, typically 50% of the initial level; Colombo & Bundy, 1983; Hunter et al., 1983; Hunter et al., 1982; Lasky, 1980; Roder et al., 2000; Rose et al., 1982). In line with the optimal-level and the two-factor models, incomplete habituation leads to a preference for processing familiar stimuli, while more complete habituation leads to a preference for processing novel ones. In support of the dual-process theory, other infant studies demonstrated that more complex stimuli generally produce sensitization (i.e., increases in looking) at early points during repetitive stimulus sequences (Bashinski et al., 1985; Colombo et al., 1997; Kaplan & Werner, 1987; Peterzell, 1993).

The differential subjective preference for novel stimuli as a function of the level of exposure has been observed with infants and adults (Montoya et al., 2017). To our knowledge, visual exploration has not yet been examined in adults. This is despite the fact that all theoretical models postulate that this represents a general learning mechanism, and thus it should be present across the life span. By combining a visual exploration task of novel and familiar pictures with an explicit evaluation task, the current study examines the roles of response to novelty and subjective preference in visual exploration. Hence, the first goal of this study was to determine whether novelty exploration in adults also depends on the level of habituation, as observed in babies. If so, we should observe that short exposure leads to exploratory behavior towards familiarity, and that longer exposure leads to novelty exploration.

The second goal was to evaluate participants’ attitude towards the stimuli to which they habituated. According to the mere exposure effect, we should observe that the more a picture is presented, the more participants tend to consider it positive, and thus prefer it. According to the optimal-level of arousal and the two-factor model of mere exposure, this should be true only up to a certain level of exposure (see Table 1).

**Table 1:** Summary of our main theoretical frameworks and the resulting hypotheses

Theoretical framework	Hypotheses
For exploratory behaviours:	
According the optimal-level, the two-factor, and the dual-process models	Short exposure leads to familiarity exploration Longer exposure leads to novelty exploration
According to the mere exposure effect	The more a picture is presented, the more participants tend to consider it positive, and thus prefer it
For subjective evaluation:	
According the optimal-level, the two-factor, and the dual-process models	The semi-familiar pictures should be considered more positively.
According to the mere exposure effect	The more a picture is presented, the more participants tend to consider it positive, and thus prefer it

## Method

### Participants

A sample of 46 French-speaking Canadian adults (29 women, 17 men, mean age = 24.9 years, *SD* = 4.60 years) participated in this study. Participants were recruited through email from a participant bank within our research group. One participant was excluded from the analyses due to equipment failure. Participants received a 5dollars (CA) compensation for their participation.

### Apparatus

Participants were seated in a soundproofed cubicle, with their eyes approximately 60 cm away of a computer screen (60 × 34 cm, 1,920 × 1,080 pixels). The monitor was placed on a table covered with a black cloth 60 cm away from the participant so that the eye tracker could successfully capture their eyes. Gaze data were collected using a Tobii X120 eye tracker, at a sampling rate of 60 Hz. Experimental equipment was operated from outside of the cubicle. All stimuli were displayed full screen and are available in an open repository (see data availability statement).

### Stimuli

Stimuli were images of two different types: fractals and *Passiflora* flowers. These types were selected because they were expected to be unfamiliar to participants, and because they contained a lot of details. We first chose fractals, because these stimuli are not present in the everyday environment, and have been used in previous studies on aesthetic preferences (Spehar et al., 2003; Spehar et al., 2016; Spehar & Taylor, 2013). We created our own images using Multibrot explorer software (Parisé, n.d.). No participant had previous experience with fractals. For flowers, two different species of the genus *Passiflora* were chosen as stimuli, because this genus has a pantropical distribution and do not naturally grow in Canada. We used available images from the internet.

**Fig. 1:**
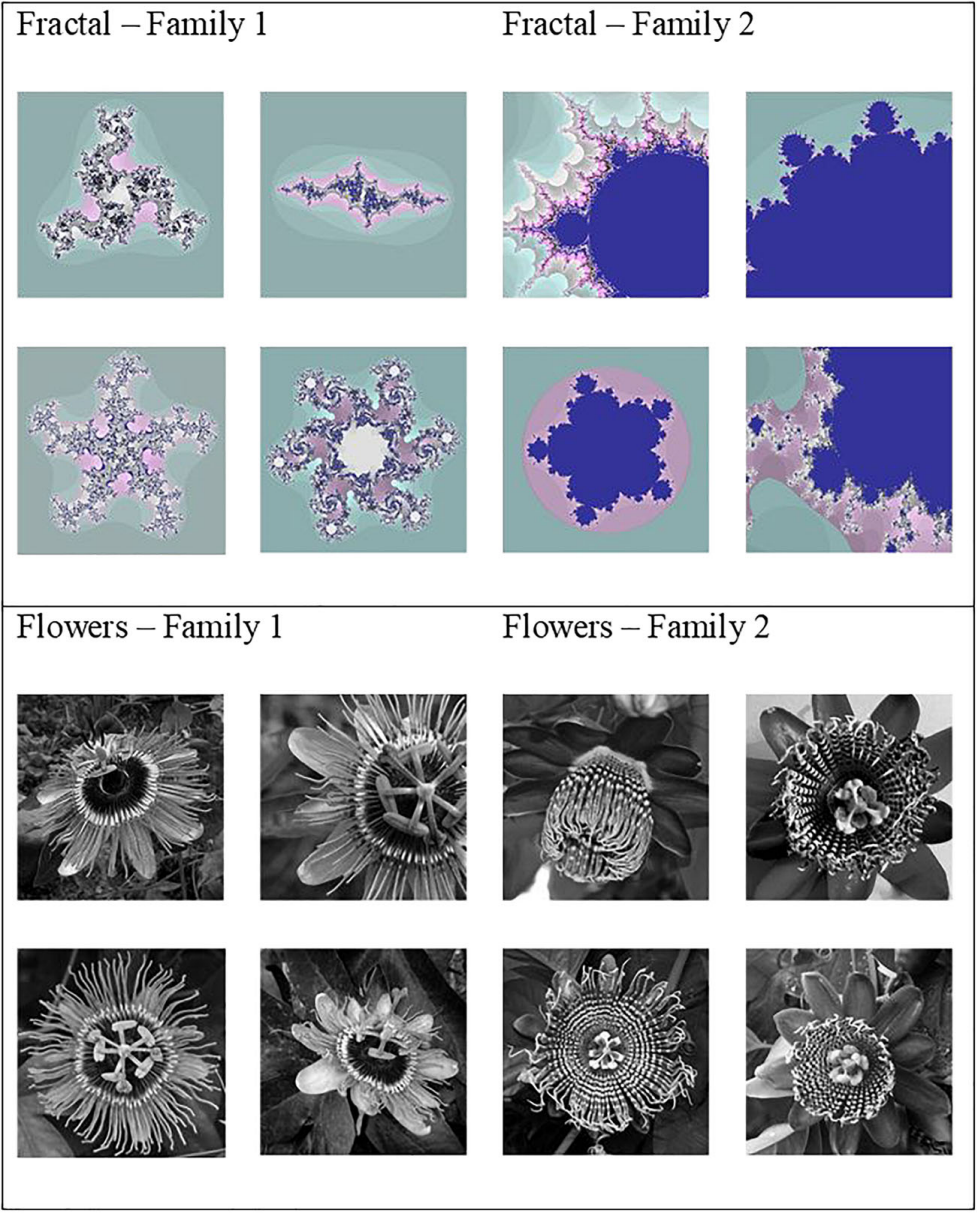
Examples of stimuli

We used two families for each type of image: “Mandelbrot” and “Julia” sets for the fractals, and “Purple haze” and “Alata” for the *Passiflora* flowers (see Fig. 1). Image families (containing 15 images each) were used instead of simple images to avoid very fast habituation, which would have made data on “short exposure” difficult to obtain and then difficult to compare with “longer exposure” ones. In addition, original colors of the fractal pictures were modified to homogenize families and thus avoid their discrimination based solely on color features. The flower pictures were transformed into gray scale images.

### Procedure

**Fig. 2:**
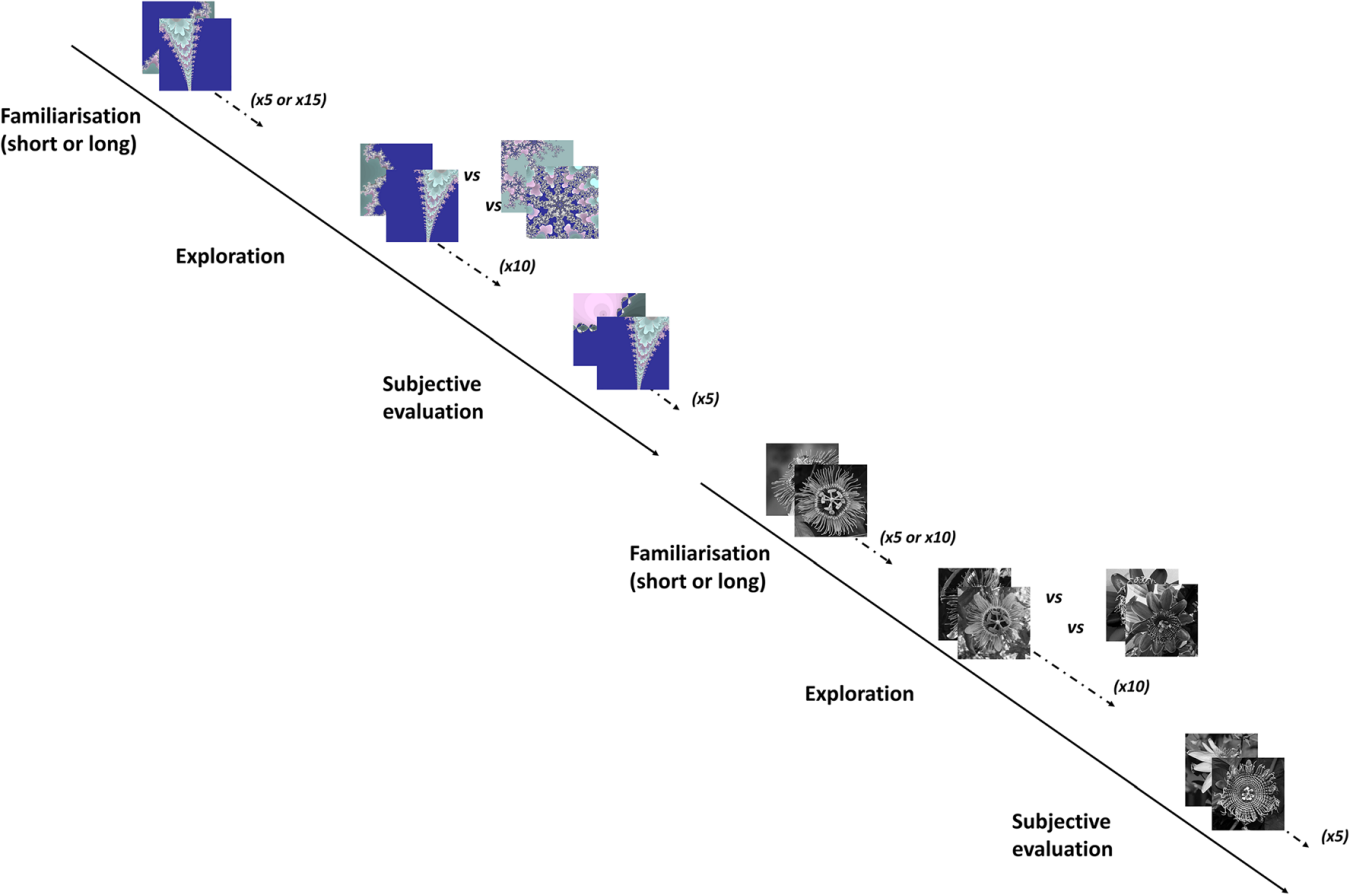
Experimental procedure. Each participant randomly started either with the flowers or with the fractals, and with either short or long familiarization, also randomly

Participants were randomly exposed with one type of stimuli first (flowers or fractals). Then, the procedure was repeated with the other type of stimuli (fractals if flowers first, or flowers if fractals first; see Fig. 2).

#### Habituation phase

We used a familiarization paradigm to generate long and a short habituation with fixed trial durations and a fixed number of exposure trials (Aslin, 2007). After a 5-point calibration procedure, the experiment started with either a short familiarization phase or a long familiarization. Participants were instructed to merely observe the images that would be displayed. Each presentation lasted 5 seconds. Short familiarization consisted of five presentations of different images from the same family, and long familiarization consisted in 15 presentations from the same family, using five different images randomly displayed three times.

#### Exploration task

The habituation phase was followed by a visual exploration phase. Again, participants were instructed to merely observe a sequence of images. Two images were presented at the same time: one novel image taken from the familiar family and one image from a novel family for 5 seconds. This was repeated 10 times. The side where each family was presented was randomized for each presentation. An eye tracker recorded looking time for each image, which allowed to calculate the proportion of time spent looking at the novel family of images.

#### Subjective evaluation task

The visual exploration phase was immediately followed by a subjective evaluation of five images: one familiar and one novel image from the two families seen during the exploration task (i.e., the familiar one seen during familiarization and exploration phases; and a “semi-familiar” one only seen during the exploration phase), and one novel image from a totally novel family. One by one, the images were presented in random order, and participants had to report their attitude towards them on a scale from 0 (*negative*) to 9 (*positive*), where 5 was considered neutral. Participants were asked to answer as quickly as possible by pressing the corresponding button on the computer keyboard. The mere exposure effect is the result of an implicit attitude. If participants have ample time to evaluate the images, automatic judgment may be inhibited and the mere exposure effect may not be as effective (Courbet, 2003).

The same procedure was then repeated with the other type of image and the other familiarization length. For instance, if a participant first saw fractals during a short familiarization phase, he/she was then presented flowers for a long familiarization phase. So, in this case, each participant would have watched 20 presentations (five familiarization, 10 explorations, five evaluations) followed by 30 presentations (15 familiarization, 10 explorations, five evaluations);

#### Data coding and analysis plan

The family familiarity was coded as follow:

- The family participants were exposed to during the long familiarization and during the exploration task was considered “familiar” (25 exposures in total).
- The families participants were exposed to during the short familiarization and during the exploration task (15 exposures), or during the exploration task only (10 exposures). were considered “semi-familiar.”
- The family participants were exposed to only during the subjective evaluation task was considered “novel” (one exposure).

The eye tracker registered gaze position at a sampling rate of 60 Hz during the visual exploration phase. The ratio between samples where the participant was looking at either stimulus was calculated to determine the proportion of stimulus looking time towards novelty. The higher this number is, the more the exploratory behaviour is oriented towards novelty. We first conducted an analysis of variance (ANOVA) to investigate the effect of stimulus type and familiarization length on the looking ratio.

We then used linear mixed-effects (LME) models to examine the effects of stimulus type (flowers versus fractals), familiarization length (short versus long), family familiarity (familiar versus semi-familiar versus novel), and image novelty (already seen versus novel) on the subjective preference ratings. All independent variables were categorical. Participant identity, visual stimuli, and order of presentation were included as random effects in LME models as each participant responded repeatedly to the diverse stimuli. Initially, all explanatory variables and the two-way interactions were fitted in a maximal model. Then, nonsignificant interactions and main terms were dropped sequentially to simplify the model. We used linear mixed models because of our unbalanced designed (we could not have familiar “already seen” images of novel families). Additional LME models were fitted to investigate significant two ways interactions. All LME models were fitted using R (Version 3.5.0; R Core Team, 2018).

## Results

**Fig. 3:**
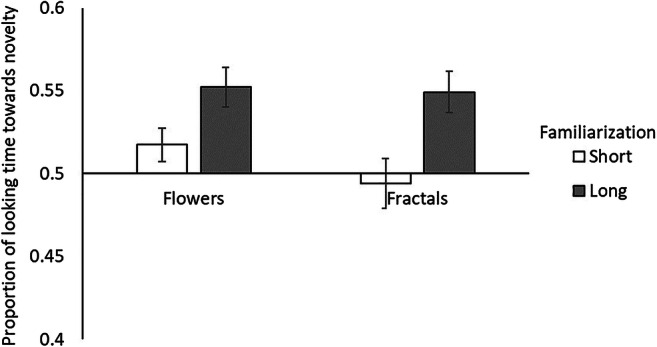
Proportion of looking time towards novelty (mean ± *SEM*) according to the type of image and familiarization length

The mean proportion of looking time towards novelty, according to the type of image and familiarization length, are presented in Fig. 3. The interaction between type of image and familiarization length did not reach significance, *F*(1, 45) = 0.679, *p* = .410, η_p_^2^ = 0.001. No effect of the type of image was observed, *F*(1, 45) = 1.137, *p* = .287, η_p_^2^ = 0.001. The length of familiarization had a significant effect on the proportion of time looking towards novelty, with relatively longer looking time after a long familiarization compared with a shorter one *F*(1, 45) = 13.239, *p* < .001, η_p_^2^ = 0.014.

We also compared the proportion of novelty-looking to chance with one-sample *t* tests. After a short familiarization, participants did not look at novel flowers, *t*(23) = 1.355, *p* = .189, or fractals, *t*(21) = −0.905, *p* = .376, more than would be expected by chance. However, following long familiarization, participants were significantly more likely to look at novel flowers, *t*(21) = 4.038, *p* = .001, and fractals, *t*(23) = 3.266, *p* = .003, than would be expected from chance.

### Subjective preference

Results from LME models are summarized in Table 2. Family familiarity tended to have an effect on participants’ attitude towards the stimuli. Indeed, pairwise comparisons showed that ratings tended to be more positive for semi familiar families (*M* = 5.652 ± 1.955) compared with familiar one (*M* = 5.282 ± 1.865, *p* = .086).

**Table 2:** Summary of the LMEs testing the effect of stimuli type, familiarization length, family familiarity and image novelty on participants’ attitude towards the stimuli

Fixed effects (order as random effect)	Estimates	*SE*	*t*	*p* value
Full model				
Intercept	5.64	0.544	10.364	<.001
Stimuli type	−0.537	0.627	−0.856	.392
Familiarization length	−0.612	0.415	−1.473	.142
Family familiarity (Semi-Familiar)	0.459	0.465	0.987	.324
Family familiarity (Novel)	0.194	0.684	0.284	.777
Image novelty	0.107	0.632	0.17	.865
Stimuli type × familiarization length	0.506	0.508	0.996	.323
Stimuli type × family familiarity (Semi-Familiar)	0.359	0.511	0.703	.482
Stimuli type × family familiarity (Novel)	−1.088	0.777	−1.4	.165
Stimuli type × image novelty	0.576	0.383	1.505	.133
Familiarization length × family familiarity	0.243	0.505	0.48	.631
Familiarization length × image novelty	−0.013	0.464	−0.027	.978
Family familiarity × image novelty	−0.827	0.54	−1.531	.126
Reduced model				
Intercept	5.010	0.293	17.121	<.001
Image Novelty	0.394	0.379	1.039	.299
Family familiarity (Semi-Familiar)	0.888	0.31	2.863	.004
Family familiarity (Novel)	−0.055	0.411	−0.134	.893
Family familiarity × image novelty	−0.837	0.44	−1.902	.058

Although that effect was not significant, the interaction between the family familiarity and image novelty was a potentially important factor affecting ratings. Further analyses on “already seen” and “novel” images separately showed that, for “already seen” images, semi-familiar families had significantly higher ratings (*M* = 5.899 ± 1.923) compared with the familiar ones (*M* = 5.065 ± 1.948, ${\upchi}_1^2$=7.327, *p* = .007). In contrast, family familiarity had no effect for “novel” images (${\upchi}_2^2$ = 0.061, *p* = .97).

## Discussion

The findings of this study concerning exploratory behaviours and subjective evaluations in adults generally support the predictions of the optimal-level, the two-factor, and the dual process models. How the models fare in relation to the research hypotheses is summarized in Table 3. The first goal of this study was to determine if, for adults, the level of habituation influences visual exploration behaviour towards a stimulus. More specifically, the aim was to ascertain whether, as with babies, short habituation is associated with visual exploration towards familiarity, whereas longer habituation leads to an exploration towards novelty. Indeed, we found that gaze was more oriented towards novelty after a long exposure, which is in line with the predictions of the optimal-level and the two-factor model.

**Table 3:** Summary of the main theoretical frameworks, the resulting hypotheses, and the validation from the study results

Theoretical frameworks	Hypotheses	Validation from results
For exploratory behaviours:		
According the optimal-level, the two-factor, and the dual-process models	Short exposure leads to familiarity exploration.	✕
	Longer exposure leads to novelty exploration.	✓
According to the mere exposure effect	The more a picture is presented, the more participants tend to consider it positive, and thus explore it.	✕
For subjective evaluation:		
According the optimal-level, the two-factor, and the dual-process models	The semi-familiar pictures should be considered more positively.	✓
According to the mere exposure effect	The more a picture is presented, the more participants tend to consider it positive, and thus prefer it.	✕

However, short familiarization did not bias orientation towards neither novelty nor familiarity. It is possible that participants were transitioning from a visual exploration toward familiarity to a visual exploration towards novelty, but our method did not allow to assess this possibility. There is a temporally limited windows for observing orientation biased towards familiarity, and our short familiarization may have been in fact too long to observe it. This could have been avoided by using a participant-controlled habituation paradigm, where the number of exposures would have been controlled by eye-tracker data and would have depended on the decline in looking time for each participant (Aslin, 2007). We cannot conclude that adults tend to look more at familiar stimuli until habituation is reached, but we can conclude that, with longer exposure, adults tend to explore new stimuli. The pattern of findings is consistent with what has been noted in human infants (Sirois & Mareschal, 2004).

The second goal was to evaluate the influence of habituation to participants’ subjective evaluation of the stimuli, and if that evaluation would follow the predictions made by the optimal-level of arousal and the two-factor model of mere exposure. Interestingly, semi-familiar families received a higher rating than familiar families. This is in line with the predictions of the optimal-level of arousal and the two-factor model of mere exposure, stating that a positive attitude should appear only once a particular level of arousal, or novelty, is reached. Since this effect of familiarity was only marginally significant, this finding should be approached with caution. However, in a similar vein, semi-familiar families received the highest ratings when participants were viewing already seen images. This is again in line with the optimal-level of arousal and the two-factor model of mere exposure.

Interestingly, a recent meta-analysis suggests that the mere exposure effect could be an artefact appearing when a sufficiently high level of familiarization is not reached (Montoya et al., 2017). Thus, orientation towards novelty after familiarization could be a means to get closer to one’s optimal level of arousal, in line with the theory of the same name. Such orientation could then also be an artefact appearing, this time, when an excessive level of familiarization is reached. In fact, novelty, along with complexity, intensity, salience or affective content, is a determinant of arousal (Bradley, 2009; Calvo et al., 2007; Faw & Nunnally, 1968; Ronga et al., 2012). Future studies could disentangle between the effect of the optimal-level of arousal (or stimulation) model and the two-factor model of mere exposure by taking physiological measures of participants and see whether their preferences correlate with their physiological arousal level, and how these measures would fit with the inverted-U shaped characteristic of both models. As we did not control for image luminance in our study, our data were not appropriate for pupillometry analyses. Future studies replicating these first results would benefit from a design that allows for pupillometry as an onjective measure of arousal (Laeng et al., 2012).

It is also worth noting that habituation is generally slower for affective stimuli than for neutral ones (Bradley, 2009; Carretié et al., 2003). Moreover, the affective value of stimuli facilitates engagement and impairs disengagement (Machado-Pinheiro et al., 2013; Nummenmaa et al., 2006) and has been found to influence the mere exposure effect (Courbet, 2003; Jin & Luo, 2011). Although some participants may have attributed an emotional valence to the flowers or the fractals (Rogowitz & Voss, 1990), the images we used were globally considered mostly neutral (ratings slightly higher than 5 on a 9-point scale). It is possible that in previous studies, a positive attitude towards the stimuli could have compensated for excessive familiarization resulting in mere exposure effect.

Altogether our study has shown that relatively long familiarization favors the exploration of novel visual stimuli, in line with previous works on habituation effects. That said, adults show a more positive attitude towards the semi-familiar stimuli compared with very familiar or very novel ones, in line with the optimal-level of arousal and the two-factor models. This study is the first to show evidence supporting both phenomena using the same set of stimuli and number of presentations, and using different dependent measures (preferential looking and subjective preference). Future neurophysiological studies may help disentangle the role of arousal and cognitive load in determining such preferences.

## Data Availability

Data and materials have been made available online in an open repository (10.17029/b5320d45-e55f-47e9-8e81-8b76a6876f1e). This study was not preregistered.
